# Housing and Health: Very Old People with Self-Reported Parkinson's Disease versus Controls

**DOI:** 10.1155/2013/710839

**Published:** 2013-03-26

**Authors:** Maria H. Nilsson, Maria Haak, Susanne Iwarsson

**Affiliations:** Department of Health Sciences, Faculty of Medicine, Lund University, P.O. Box 157, 221 00 Lund, Sweden

## Abstract

*Objectives.* To explore whether aspects of housing and health among very old people with self-reported Parkinson's disease (PD) differ from matched controls. *Methods.* Data from the ENABLE-AGE Survey Study were used to identify people with self-reported PD (*n* = 20) and three matched controls/individual (*n* = 60). The matching criteria were age (mean = 82 years), sex, country, and type of housing. The analyses targeted problems in activities of daily living, objective and perceived aspects of housing, for example, number of environmental barriers, accessibility (i.e., person-environment fit), and usability. *Results.* The number of physical environmental barriers did not differ (*P* = 0.727) between the samples. The PD sample had more (*P* < 0.001) accessibility problems than controls and perceived their homes as less (*P* = 0.003) usable in relation to activities. They were less independent and had more functional limitations (median 5 versus 2; *P* < 0.001), and 70% experienced loss of stamina or poor balance. *Conclusions.* Due to the fact that they have more functional limitations than very old people in general, those with self-reported PD live in housing with more accessibility problems. This explorative study has implications for rehabilitation as well as societal planning, but larger studies including people with a confirmed PD diagnosis are needed.

## 1. Introduction

An increasing proportion of very old people remain living in their ordinary homes despite declines in health. With an increased life expectancy for the general population and for those living with chronic diseases, this poses major challenges to rehabilitation as well as societal planning and housing development [1, 2]. Although Parkinson's disease (PD) is typical for old age, older people with PD are often excluded in research [3]. The knowledge on the life situation of those ageing with PD is therefore insufficient, and little is known about their housing and health situation. In order to develop more efficient rehabilitation strategies for those living with a chronic disease during many years, such knowledge is needed.

In PD research, most studies are based on hospital-based samples excluding old and very old people [3], with attention mainly to disease-specific outcomes. Using such selected samples with no consideration to contextual factors is quite insufficient. According to the International Classification of Functioning, Disability, and Health (ICF) [4], environmental factors influence activity and participation. Examples of physical environmental barriers in the housing environment and in its close exterior surroundings are high thresholds; wall-mounted kitchen cupboards and shelves placed extremely high, and no/too few seating places along walking paths. Environmental barriers constitute one of the two components of accessibility, a relative concept implying that accessibility problems should be expressed as a person-environment (P-E) relationship [5]. In other words, accessibility is the encounter between the functional capacity of the individual (personal component) and the design and demands of the physical environment (environmental component). Most important, the environmental component of accessibility refers to compliance with official standards for design of the built environment. Thus, accessibility is an objective concept [5]. To enable participation in life situations, the recently published World Disability Report stresses the need for focusing on accessibility issues and environmental barriers [6]. Turning to the concept of usability, it implies that a person should be able to use, that is, to move around, be in, and use, the environment on equal terms with other citizens [5]. Accessibility is a necessary precondition for usability, which takes user perceptions into account. Usability is thus mainly subjective in nature. Most important, there is a third component distinguishing usability from accessibility, that is, the activity component [5]. In order to deliver efficient interventions, rehabilitation approaches that take contextual factors into account are imperative. The majority of PD studies have addressed body functions in contrast to activity, participation, and contextual factors. Consequently, little is known about the relationship between contextual factors and activity, participation, and health factors in people with PD.

Major gerontology studies are often based on population samples applying perspectives and methodologies quite different from those of neuroscience and rehabilitation. When it comes to theoretical foundations, the model most often referred to is Lawton's ecological theory of ageing (ETA) [7, 8] in which the person is defined in terms of a set of competencies and the environment is defined in terms of press. Thus, person-environment fit (P-E fit) comprises two interactive components: the personal component and the environmental component. In the ETA, P-E fit is a prerequisite for the matching between the personal component and environmental component, denoted “adaptation.” Manifested in real life situations, performance of activities of daily living (ADL) is one important aspect of adaptation [9]. When health declines in very old age, the environmental pressure often exceeds the personal capacities, resulting in more P-E fit problems and negative health outcomes. Based on the notion of P-E fit, research on housing and health in old age considers contextual factors such as objective and perceived aspects of housing [10–12]. Besides usability, perceived aspects of housing such as housing satisfaction, meaning of home, and housing-related control beliefs are related to health in very old age [10–12]. Targeting such aspects represents a quite novel approach for PD research, and the knowledge of the situation of very old people with PD in comparison with very old people in general is virtually nonexisting. Consequently, this study aimed at exploring aspects of housing and health in ordinary housing among very old people with self-reported PD as compared to matched controls. The following general hypotheses guided the study.

- Since ordinary housing displays great diversity in design, there is no reason to believe that very old people with PD live in dwellings with a different number of environmental barriers than very old people in general.
- There is reason to assume that very old people with PD have more functional limitations and use mobility devices to a greater extent than very old people in general, resulting in more housing accessibility problems.
- Since usability considers activity performance, it might be rated lower by very old people with PD than by very old people in general. As to other perceived aspects of housing, there is less reason to assume that very old people with PD differ from others.

## 2. Participants and Methods 

### 2.1. Project Context

This study is based on data from the ENABLE-AGE Survey Study [10], gathered in Sweden, Germany, the United Kingdom, Hungary, and Latvia. The target sample in each country was very old people (75–89 years), living in single-person households in urban areas. The total sample included 1,918 participants (78% women). The study was conducted in accordance with the Declaration of Helsinki; all participants were enrolled after informed consent, following the ethical guidelines of each country. After training, interviewers collected data at home visits [13]. Details on the ENABLE-AGE have been published elsewhere (see, e.g., [10, 12]).

### 2.2. Study Samples

The present study is a cross-sectional comparison between two subsamples retrieved from the ENABLE-AGE database; one sample of individuals, responding to structured questions based on the ICD-10, reported having PD (PD sample) versus a matched control sample (for characteristics see Table 1). Twenty-one individuals with self-reported PD were identified, but one Hungarian woman was excluded due to extensive missing data. The final PD sample consisted of 20 individuals (15 women and 5 men; mean age 82 years).

Each individual reporting having PD was individually matched with three controls [14] from the ENABLE-AGE database. The matching criteria were sex, country, age (± one year), and type of housing (Table 1). By means of the software R version 2.12.1 (R Development Core Team, 2010), the three controls were randomly selected among all individuals fulfilling the matching criteria [14]. The matched control sample included 60 individuals (45 women and 15 men; mean age 82 years).

### 2.3. Instruments

All included instruments fulfilled basic criteria for reliability and validity (for details, see publications referred to below). For project-specific instruments, an interrater reliability study [15] and psychometric analyses were accomplished [16].

### 2.4. Aspects of Housing

Objective housing was operationalized as the number of environmental barriers in the home and the magnitude of accessibility problems, assessed using the Housing Enabler (HE) [17]. With this instrument, accessibility is assessed by professionals, based on the notion of P-E fit [7]. The administration of the HE contains three steps.

**Step 1**

Representing the personal component of P-E fit: Interview and observation of a profile of functional limitations (13 items) and dependence on mobility devices (2 items: dependence on walking devices and/or wheelchair), all dichotomously assessed (no/yes). The 13 items that target functional limitations are difficulty in interpreting information; visual impairment; blindness; loss of hearing; poor balance; incoordination; limitations of stamina; difficulties in moving head; reduced upper extremity function; reduced fine motor skill; loss of upper extremity skills; reduced spine and/or lower extremity function; extremes of size and weight.

**Step 2**

Representing the environmental component of P-E fit: Observation and dichotomous assessment (no/yes) of 188 physical environmental barriers in the home and the immediate outdoor environment.  Step 2 generates the variable “number of environmental barriers.”

**Step 3**

Representing the facet of P-E fit denoted accessibility: Based on the assessments in Steps 1 and 2, by means of a complex matrix procedure with predefined 0–4 scores, a total accessibility score is computed by means of special software (for details, see [17]). Thus, the variable accessibility is operationalized as the magnitude of accessibility (P-E fit) problems caused by the case-specific combination of functional limitations/dependence on mobility devices and environmental barriers; higher scores = more accessibility problems. In cases with no functional limitations/dependence on mobility devices, the accessibility score is 0.

Perceived aspects of housing were captured by means of the four-domain model of perceived housing [16], based on self-ratings. The four domains include housing satisfaction (i.e., in relation to physical housing conditions), usability in the home, the meaning of home, and housing-related control beliefs. Housing satisfaction was assessed by using the single item: “Are you happy with the condition of your home?” with response options ranging from 1 (“definitely not”) to 5 (“yes, definitely”). With the Usability in My Home Questionnaire (UIMH), the participant evaluates the degree to which the physical housing environment supports activity performance at home. In the present study, the subscales “activity aspects” (4 items) and “physical environmental aspects” (6 items) were used. Items are rated from 0 (“not at all”) to 5 (“fully agree”); higher scores denote higher usability. The Meaning of Home Questionnaire was used to rate the physical (7 items), behavioral (6 items), and cognitive/emotional (10 items) aspects of bonding/attachment to the home. Each statement is rated from 0 (“strongly agree”) to 10 (“strongly disagree”); higher scores mirror a stronger bonding/attachment. Finally, we used the combined external control subscale (16 items, rated from 1 = “not at all” to 5 = “very much”) of the Housing-related Control Beliefs Questionnaire. External control means either that some other person, luck, chance, or fate are responsible for events or things that happen. For further details and original references to the specific instruments, see [16].

### 2.5. Aspects of Health

Representing the outcome of adaptation as manifested in real life situations [9], ADL problems were captured and assessed by professionals as well as by means of self-ratings. Independence in daily activities was assessed by professionals through interview and observation using the ADL Staircase [18]. This instrument comprises five personal ADL (P-ADL: feeding, transferring, going to the toilet, dressing, and bathing) and four instrumental ADL (I-ADL: cooking, transportation, shopping, and cleaning). The assessment captures dependence on assistance from another person during activity performance (rated as independent/partly dependent/dependent). For all ADL items scored as “independent,” the interviewer also asked a project-specific dichotomous (Yes/No) question: “Even if you manage on your own, do you experience any difficulty when performing…?” The nine items were dichotomized as follows: dependent, partly dependent or having difficulties (1)/independent, and with no difficulties (0). Perceived independence in daily activities was captured by a single item: “All in all, how would you evaluate your own independence, that is, in performing activities of daily living?” [16]. The response options range from 0 (“completely dependent”) to 10 (“completely independent”).

The variables number of functional limitations and use of mobility devices (Yes/No) were retrieved from Step 1 of the HE [17]. Self-perceived health was rated using the global item from the SF-36 [19]: “In general would you say your health is…?”, rated from 1 (“excellent”) to 5 (“poor”). Depressive symptoms were dichotomously assessed with the 15-item version of the Geriatric Depression Scale (GDS) [20].

### 2.6. Data Analysis

Internal missing was treated in the following way. If there was a missing value for one of the individuals reporting PD, all the controls belonging to that individual were also excluded from that particular analysis. If there was a missing value for one or two of the controls, the individual reporting PD and the remaining controls were included in the analysis. Missing data are reported in Tables 2 and 3.

All dichotomous variables were compared using the Mantel-Haenszel test, with continuity correction, which takes the matching with multiple controls into account. These tests were performed using SPSS Statistics 18 for Windows (IBM Corporation, Somers, NY, USA). For ordinal scores, we applied a version of Wilcoxon signed rank test extended to include the multiple controls [21]. For these tests, *P* values were obtained using Monte Carlo simulations in the R-programming environment, version 2.12.1. Given the exploratory nature of this study, no correction for multiple tests was applied; that is, results with *P* values <0.05 were considered statistically significant.

## 3. Results

### 3.1. Aspects of Housing

Regarding objective aspects of housing (Table 2), the number of environmental barriers in the home did not differ significantly (*P* = 0.727) between the two samples. Turning to accessibility (P-E fit) problems, the participants in the PD sample had significantly (*P* < 0.001) more problems than the controls; that is, the median (q1–q3) scores were 192 (112–232) versus 63 (14–28).

Regarding perceived aspects of housing (Table 2), the participants in the PD sample reported their home as less usable in relation to activities (*P* = 0.003), but the two samples did not differ (*P* = 0.600) regarding physical environmental aspects of usability. The participants in the PD sample were less attached to their home in relation to physical (*P* = 0.018) and behavioural (*P* < 0.001) aspects of meaning of home than the controls, while there was no difference (*P* = 0.444) regarding cognitive/emotional aspects (Table 2). External housing-related control beliefs (*P* = 0.867) and housing satisfaction (*P* = 0.114) showed no significant differences between the two samples (Table 2).

### 3.2. Aspects of Health

The participants with self-reported PD were more (*P* = 0.018) dependent on walking aids than the controls (Table 3), that is, 10/20 (50%) versus 12/60 (20%). Their total number of functional limitations was also significantly (*P* < 0.001) higher (Table 3). In the PD sample, the three most common functional limitations were reduced spine and/or lower extremity function (18 out of 20; 90% versus 67% in controls), poor balance (14/20; 70% versus 28% in controls), and loss of stamina (14/20; 70% versus 47% in controls); see also footnote in Table 3. In the PD sample, the vast majority of those having reduced spine and/or lower extremity function also had loss of stamina (13/18; 72%) and poor balance (12/18; 67%).

For six out of the nine ADLs, there were statistically significant differences between the two samples (Table 3) with the PD sample demonstrating more ADL problems; that is, they were more dependent or reported having more difficulties. In the activities transfer, dressing and cleaning, there were no statistically significant differences. More participants in the PD sample were dependent in all four I-ADLs (*P* = 0.011) and in all five P-ADLs, *P* < 0.001 (Table 3). Self-ratings of functional independence showed that those in the PD sample perceived themselves as less (*P* < 0.001) independent than the controls (Table 3).

In both samples, the participants rated in median their general health as “fair” (i.e., 4: *P* = 0.190), and q1–q3 were 3–5 (“good”–“poor”) for the PD sample and 3-4 (“good”-“fair”) for the control sample. The participants in the PD sample reported significantly (*P* = 0.048) more depression symptoms than the controls (Table 3).

## 4. Discussion

As stated in the general hypothesis that guided the present study, the main results indicate that very old people with self-reported PD live in housing with more accessibility problems and experience less usability of their home than matched controls. At the same time, they seem to be less attached to their home although housing satisfaction and perceived control over their housing situation do not statistically differ. Even if this exploratory study is based on a small sample of participants with self-reported PD, it rests on solid methodology [10, 12, 16, 17], well acknowledged in research on ageing. Since this type of research has yet not been seen in PD, the knowledge contribution of this explorative study is substantive.

Although the present study shows that very old people with self-reported PD live in housing situations with more accessibility (P-E fit) problems, there was no statistically significant difference between the PD sample and the controls regarding number of environmental barriers (Table 2). This highlights the fact that the accessibility (P-E fit) problems were generated by the higher number of functional limitations and more use of walking aids in the PD sample (Table 3). This is in line with previous studies (see, e.g., [12, 22]), and supports the basic notion of P-E fit as described in the ETA [7].

While seldom at target in PD research, in rehabilitation research and clinical settings an explicit attention to contextual factors is imperative; that is, focusing on accessibility issues and environmental barriers is imperative in order to enable activity and participation [6]. For example, by means of reduction of accessibility problems (i.e., by strengthening of the functional capacity of the individual or/and by removing environmental barriers), the usability of the home, manifested in self-perceived reduction of ADL problems, might be improved. In order to reduce accessibility problems among very old people with PD, our results suggest that rehabilitation primarily should target independence in walking, reduced spine and lower extremity function, limitations of stamina, and balance problems. A recent Cochrane review concluded that physical therapy significantly improves outcomes of walking, mobility, and balance as compared with no intervention [23]. The treatment effects were, however, generally small and the majority of studies had a short follow-up period. It should be noted that there is a lack of rehabilitation studies that target very old people with PD. This is highly warranted due to the increased life expectancy of the general population and for those living with chronic diseases, but also since older people with PD are often excluded in research [3].

Our finding that very old people with self-reported PD live in housing with more accessibility problems should be seriously considered, not the least since people with PD have an increased risk for falls even at a younger age [24]. Thus, the P-E fit perspective is highly relevant for research and fall prevention in PD. According to a previous study [22], accessibility (P-E fit) problems are more common among very old people who fall than among nonfallers, and also a stronger predictor for falls than the number of environmental barriers per se. This suggests that fall prevention in the home should target P-E fit and not only environmental barriers, with a specific attention on strengthening the functional capacity of the individual, that is, the personal component of P-E fit. A prior study showed that the most common functional limitations in very old people were reduced spine and/or lower extremity function, limitations of stamina, and poor balance [25]. Although the same top three functional limitations were identified for the PD sample, the prevalence was higher among very old people with self-reported PD than for matched controls (e.g., poor balance: 70% versus 28%). Consequently, individualized approaches might be more efficient than removing environmental barriers based on traditional fall risk hazard checklists; that is, based on a decomposition of the individual accessibility (P-E fit) score generated by the HE [17], fall risks could be eliminated by a case-specific, targeted elimination of the environmental barriers that in relation to the individual profile of functional limitations generate the most severe accessibility (P-E fit) problems. Such an analysis is easily accomplished by means of the HE software. However, it should be noted that based on the definition of accessibility (P-E fit) [7] that underpins the HE [17], physical environmental barriers are assessed based on standards and guidelines for housing design. Removing barriers based on these might not be sufficient from the individual perspective; that is, the housing design features specified in the existing standards and guidelines may nevertheless generate problems for individuals with more complex profiles of functional limitations [26]. Therefore, more research is needed on housing accessibility among people with neurological disorders.

Further, the present results suggest that very old people with PD are less attached to their home in physical and behavioural aspects than very old people in general. Earlier studies show that a strong bonding to home facilitates coping with ADL difficulties [16], whereas a weak bonding relates to a higher magnitude of accessibility problems [27]. In other words, in the present study those reporting having PD were probably less attached to their home due to having more accessibility problems [27]. If reducing the accessibility problems in their housing situation, it may be speculated that a stronger bonding to home could strengthen their ability to cope with ADL difficulties [16].

Regarding aspects of health, the results are as could be expected, and thus in line with what we hypothesized; that is, very old people with self-reported PD are less independent, have more functional limitations, and are more dependent on walking aids (Table 3). This is not surprising since gait and balance problems (including falls and fear of falling) are common among people with PD [24, 28, 29], and ADLs are affected already in de novo PD [30]. Demonstrating the relevance of studying aspect of housing and health among people with PD, it should be noted that dependence on others for ADL has been shown to be a predictor of relocation to assisted living [31]. This is of specific importance since people with PD are more likely to be placed in assisted living, causing high costs to society [1].

There are study limitations and challenges that need to be kept in mind when interpreting our results. First of all, the population-based sample was based on self-reported PD, and, consequently, we lack clinical data describing PD severity and disease-specific problems. We do, however, have data on functional limitations, serving as an indirect indicator of disease severity. Since PD-specific factors may negatively influence P-E fit, these ought to be addressed in future studies. The individuals that reported having a PD diagnosis (mean age 82 years) constituted about 1% of the original cross-national database (*n* = 1,918). For this age group, this PD prevalence is low [32]. Since people with PD are admitted to assisted living at an earlier age than the general population [1], the low prevalence rate probably reflects the inclusion criteria of the original study (i.e., single living in ordinary housing in urban areas) [10]. There were some prevalence differences among the national samples, ranging from one (Sweden) to seven individuals (Hungary). Since the ENABLE-AGE Project targeted ordinary housing, a plausible explanation may be cultural or societal differences among the countries, for example, differences in the availability of housing options such as assisted living facilities. These are mere speculations but highlights one type of challenges in cross-national research [13]. Cautious interpretation is needed since there were few individuals with self-reported PD in each of the national samples. The original sampling imposes some additional concerns for the external validity of our findings. It targeted a selected portion of people in very old age and does not represent the population in general, but rather a healthier segment in a European context. Furthermore, reflecting the dominance of women in the very old population, it consisted of 78% women explaining the female preponderance in the present study, despite that PD is more common among men [32]. Another important consideration is that, due to the exploratory nature of the study, we did not correct for multiple comparisons. This calls for cautious interpretation of our findings, especially so for results not showing highly significant *P* values such as for depression (*P* = 0.048) and some of the results regarding ADL, for example, transfer (*P* = 0.047) and shopping (*P* = 0.035) (Table 3). Turning to a strength of the study, due to the large scale study database available, we were able to apply a strong design by selecting three randomized matched controls per case [14]. Notwithstanding the limitations discussed, this study demonstrates that important knowledge can be gained by fusing different strands of research traditions. The findings can be used as a starting point for larger prospective studies, with optimized validity for those ageing with PD. Future prospective studies that target accessibility problems in PD should preferably also include variables that tap PD-specific problems and symptoms, for example, gait problems and freezing of gait. In addition, future PD studies ought to investigate which environmental barriers that account for the most accessibility problems.

## 5. Conclusions

The results of this explorative study suggest that despite similar housing environments in terms of number of environmental barriers, very old people with self-reported PD live in housing with more accessibility (P-E fit) problems than very old people in general. The accessibility problems are generated by their higher number of functional limitations and more use of walking aids. As a consequence, very old people with self-reported PD also have more ADL problems. Since they perceive their homes as less usable in relation to activities and seem to be less attached to their home, the results are relevant for issues such as fall prevention and relocation counselling. This study demonstrates that research on housing and health among people with self-reported PD has potential to generate knowledge of importance for the development of rehabilitation and societal planning for this patient group. Larger prospective studies including people with a confirmed PD diagnosis are, however, needed to support or refute the present findings.

## Figures and Tables

**Table 1:** Sample characteristics: very old people reporting having Parkinson's disease (PD) (*n* = 20) and controls not reporting having PD (*n* = 60).

Variable	PD, *n* = 20	Controls, *n* = 60
Age, mean (SD, min–max)^a^	82 (3.6, 76–90)	82 (3.6, 76–91)
Sex, *n* women/men (% women)^a^	15/5 (75)	45/15 (75)
Country, *n* (%)^a^		
Germany	4 (20)	12 (20)
Hungary	7 (35)	21 (35)
Latvia	4 (20)	12 (20)
Sweden	1 (5)	3 (5)
United Kingdom	4 (20)	12 (20)
Type of housing, *n* (%)^a^		
Multidwelling block	16 (80)	48 (80)
One-family house	2 (10)	6 (10)
Semidetached/two-family house	1 (5)	3 (5)
Other	1 (5)	3 (5)

^a^Matching variable.

**Table 2:** Comparison of aspects of housing between very old people reporting having Parkinson's disease (PD) (*n* = 20) and controls not reporting having PD (*n* = 60).

Variable	PD, *n* = 20		Controls, *n* = 60		
	Median	q1–q3	Median	q1–q3	*P* value^a^
Number of environmental barriers, (HE)	48	36–61	52	34–63	0.727
Accessibility, (HE)	192	112–232	63	14–128	<0.001
Usability (UIMH)					
(i) Activity aspects^b,c^	3.8	3-5	5	4.3–5	0.003
(ii) Physical environmental aspects^b^	4.1	3.4–4.8	4.3	3.6–5	0.600
Meaning of home (MOH)					
(i) Physical bonding^b,c^	7	5.3–8.9	8.6	6.9–8.9	0.018
(ii) Behavioral bonding^c^	7	4.8–8.6	8.8	7.3–10	<0.001
(iii) Cognitive, emotional bonding^b,c^	8	7.1–9.4	8.2	7.4–9	0.444
External housing-related control beliefs (HCQ)^b,c^	2.7	2.1–3.4	2.8	2.1–3.2	0.867
Housing satisfaction^c^	5	4-5	5	4-5	0.114

Decimals are only given when rounding was needed.

^a^A version of Wilcoxon signed rank test extended to include multiple controls was used.

^b^For these variables the PD sample had missing values (for 1 to 2 participants); the number of controls was reduced accordingly.

^c^For these variables some of the controls had missing values (for 1 to 4 participants).

HE: Housing Enabler; higher accessibility scores mean more accessibility (person-environment fit) problems (range 0 to >2000; theoretically but never reached in reality). UIMH: Usability In My Home; higher scores are positive. MOH: Meaning of Home Questionnaire; higher scores mean stronger attachment to the home. HCQ: Housing-Related Control Beliefs Questionnaire; higher scores indicate more external control. Housing satisfaction is rated from 1 (no, definitely not) to 5 (yes, definitely).

**Table 3:** Comparison of aspects of health between very old people reporting having Parkinson's disease (PD) (*n* = 20) and controls not reporting having PD (*n* = 60).

	PD, *n* = 20		Controls, *n* = 60		
	*n *	%	*n *	%	*P* value^a^
ADL Staircase item^b^					
(1) Feeding	7/20	35	2/60	3	0.001
(2) Transfer	10/20	50	13/60	22	0.047
(3) Toileting	7/20	35	3/60	5	0.003
(4) Dressing	10/20	50	15/60	25	0.074
(5) Bathing	14/20	70	23/60	38	0.017
(6) Cooking	12/20	60	15/60	25	0.017
(7) Transportation	14/18	78	21/53	40	0.017
(8) Shopping	16/19	84	29/57	51	0.035
(9) Cleaning	17/19	90	41/57	72	0.206
Personal ADL (items 1–5)^b,c^	6/20	30	0/60	0	<0.001
Instrumental ADL (items 6–9)^b,c^	9/18	50	9/53	17	0.011
Dependence on walking aids (HE)	10/20	50	12/60	20	0.018

	Median (q1–q3)		Median (q1–q3)		

Self-rated functional independence^d^	5 (5–7)		8 (7–10)		<0.001
Number of functional limitations (HE)^e^	5 (4–7)		2 (1–4)		<0.001
Depression (GDS)^f^	6.5 (3.1–9.8)		4 (2.1–6.9)		0.048

^a^Dichotomous variables were compared using the Mantel-Haenszel test, with continuity correction. Sum-scores/ordinal variables were compared using a version of Wilcoxon signed rank test extended to include multiple controls.

^b^Assessed as dependent or partly dependent, or reporting having difficulties.

^c^The results refer to being dependent or reporting having difficulties in all personal or instrumental ADLs, respectively.

^d^Higher scores are “better,” that is, more independent.

^e^In both samples, the three most common functional limitations were reduced spine and/or lower extremity function, loss of stamina, and prevalence of poor balance.

^f^Higher scores are “worse,” that is, more depression.

ADL: Activities of Daily Living; HE: Housing Enabler; GDS: Geriatric Depression Scale.
